# Mitogenome Characterization and Phylogenetic Insights Into Blind Mole Rats, *Nannospalax nehringi,* and *N. turcicus*, From Türkiye

**DOI:** 10.1002/ece3.73989

**Published:** 2026-07-10

**Authors:** Barış Yıldız, Ahmet Yesari Selçuk, Saffet Teber, Mehmet Baran, Yüksel Coşkun, Coşkun Tez, Osman İbiş

**Affiliations:** ^1^ Life Sciences and Technologies Application and Research Center Kafkas University Kars Türkiye; ^2^ Department of Forestry, Artvin Vocational School Artvin Çoruh University Artvin Türkiye; ^3^ Department of Agricultural Biotechnology, Faculty of Agriculture Eskisehir Osmangazi University Eskisehir Türkiye; ^4^ Department of Agricultural Biotechnology, Graduate School of Natural and Applied Sciences Erciyes University Kayseri Türkiye; ^5^ Department of Biology, Faculty of Sciences Dicle University Diyarbakır Türkiye; ^6^ Department of Biology, Faculty of Sciences Erciyes University Kayseri Türkiye; ^7^ Genome and Stem Cell Center (GENKOK) Erciyes University Kayseri Türkiye

**Keywords:** mitogenome, *N. nehringi*, *Nannospalax turcicus*, phylogeny, Spalacidae, Türkiye

## Abstract

Blind mole rats (BMRs) are subterranean rodents adapted to hypoxic environments and increasingly recognized as biomedical models. In this study, the complete mitochondrial genomes of *Nannospalax turcicus* (16,421 bp) and *N. nehringi* (16,424 bp) were sequenced and analyzed phylogenetically. Both mitogenomes displayed conserved gene content and order. Phylogenetic relationships within the family *Spalacidae* were inferred using mitochondrial genomes. Bayesian and Maximum Likelihood analyses produced congruent topologies supporting subfamily monophyly. Codon usage‐based clustering yielded a dendrogram consistent with the phylogenetic tree topologies. *N. turcicus* and *N. nehringi* formed a well‐supported monophyletic clade, clearly separated from other *Nannospalax* species. Genetic divergences between *N. nehringi* and *N. turcicus* were 9.26% and 11.77% based on mitogenomic and cytochrome *b* (*CYTB*) data, respectively. *CYTB* sequence comparisons revealed low divergence (0.75%) within the Thracian *N. leucodon* populations, whereas a notably higher divergence (4.6%) was observed between these and the Çanakkale specimens currently annotated as *N. turcicus*, indicating marked regional genetic structuring across the Bosphorus/Dardanelles barrier. Additionally, genetic divergence between two *N. nehringi* samples from Kars was 1.96%. *CYTB* sequence analysis, a clear mitochondrial divergence was observed between *N. turcicus* specimens and *N. leucodon* specimens, indicating possible genetic differentiation between the BMR populations within the Turkish Thrace region. These findings highlight substantial mitochondrial differentiation and emphasize the need for broader sampling and integrative taxonomic approaches.

## Graphical abstract

This study presents the complete mitochondrial genomes of *Nannospalax nehringi* and *N. turcicus* from Türkiye and evaluates their phylogenetic relationships within Spalacidae. Both genomes exhibited conserved structure, and phylogenetic analyses using ML and BI approaches supported subfamily monophyly and a well‐supported clade for the two species. Genetic divergence and Cytb analyses revealed notable intraspecific variation and regional structuring, highlighting the need for broader sampling and integrative taxonomic approaches.

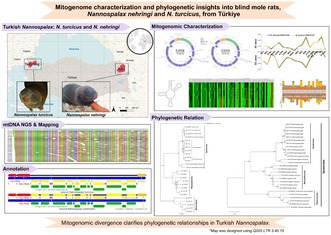

## 1 Introduction

Blind mole rats (BMRs), subterranean rodents of the family Spalacidae, represent a compelling model for studying chromosomal evolution, speciation, and physiological adaptation to hypoxic underground environments. Distributed across southeastern Europe, Asia Minor, the Caucasus, the Middle East, and North Africa, BMRs spend their entire lives in sealed, self‐constructed burrow systems and have undergone extensive evolutionary modifications, including total blindness and hypoxia tolerance (Bugarski‐Stanojević et al. 2022; Schmidt et al. 2017; Topachevskii 1969). Owing to these unique adaptations, BMRs have recently gained attention as emerging model organisms in biomedical fields, particularly in cancer resistance research (Ashur‐Fabian et al. 2004; Odeh et al. 2023, 2020; Schülke et al. 2012; Yıldız et al. 2021).

BMRs are classified into two genera within the subfamily *Spalacinae*: *Spalax* and *Nannospalax*. The genus *Nannospalax*, endemic to southeastern Europe, Anatolia, and the Middle East, has drawn considerable attention due to its extensive chromosomal variability and cryptic morphology (Bugarski‐Stanojević et al. 2022). In Türkiye, the genus *Nannospalax* remains taxonomically contentious and is currently represented by three superspecies: *N. leucodon* extends from Central Europe into Thrace, *N. nehringi* is distributed widely across Anatolia, and *N. ehrenbergi* spans southern Anatolia, the Middle East, the eastern Mediterranean, and North Africa (Karataş et al. 2021; Kryštufek and Vohralík 2009; Sözen and Çolak 2025; Wilson and Reeder 2005). In Türkiye, these three superspecies have further diversified into at least six distinct taxa: *N. turcicus*, *N. nehringi* , *N. cilicicus*, *N. tuncelicus*, *N. leucodon* , and *N. ehrenbergi* , as supported by an integrative body of evidence from molecular studies (Bugarski‐Stanojević et al. 2022, 2020; Coşkun and Ulutürk 2004; Hadid et al. 2012; Kankiliç et al. 2005; Kankılıç, Civelek, and Köse 2024; Kankılıç, Çelikbilek, et al. 2024; Kryštufek et al. 2012; Németh et al. 2024).

The restricted dispersal capacity of subterranean mammals is thought to facilitate the emergence of evolutionarily isolated populations and the accumulation of chromosomal rearrangements (Bush et al. 1977). The genus *Nannospalax* exhibits exceptional karyotypic diversity across its fragmented distributional range, primarily driven by Robertsonian fissions and fusions, pericentric inversions, and centromeric shifts (Arslan et al. 2016; Arslan and Zima 2014; Nevo et al. 1995, 1994; Wahrman et al. 1969a, 1969b). *Nannospalax* populations in Türkiye exhibit remarkable karyotypic diversity, with diploid chromosome numbers ranging from 2n = 36 to 62 (Arslan et al. 2016). This exceptional chromosomal variation has stimulated numerous molecular genetic studies aiming to resolve taxonomic uncertainties, assess species boundaries, and explore evolutionary processes (Bugarski‐Stanojević et al. 2022; Hadid et al. 2012; Kankılıç, Civelek, and Köse 2024; Kankılıç, Çelikbilek, et al. 2024; Kryštufek et al. 2012; Németh et al. 2024; Arslan et al. 2010; Kandemir et al. 2012; Kankilic and Gürpınar 2014; Matur et al. 2019). In these studies, mitochondrial DNA regions such as *COX1*, *cytochrome b* (*CYTB*), *12S* and *16S rRNAs*, and the *D‐loop* have been widely used to infer phylogenetic relationships among chromosomally distinct populations. However, these traditional markers often lack the resolution needed to disentangle complex evolutionary histories, particularly in cryptic and rapidly diverging subterranean lineages. Recent advances in next‐generation sequencing technologies have enabled the generation of complete mitochondrial genomes, which offer significantly improved phylogenetic resolution and provide new insights into mitochondrial genome evolution and species diversification in subterranean mammals (Fabre et al. 2017; Li, Shi, et al. 2016).

In most animals, the mitochondrial genome is a compact DNA molecule ranging from 15 kb to 20 kb in length and typically encodes a conserved set of 37 genes. These genes include 13 PCGs (protein‐coding genes), 22 *tRNA*s (transfer RNA genes), and 2 rRNAs (ribosomal RNA genes: *12S rRNA* and *16S rRNA*). Despite minor variations in some taxa, this gene composition is highly conserved across the animal kingdom (J. L. Boore 1999). Complete mitogenome sequences have increasingly been employed as robust molecular markers in phylogenetic, population genetic, and evolutionary studies of mammals (de Abreu‐Jr et al. 2020; Demirtaş et al. 2022; İbiş et al. 2023, 2022, 2020; Lamelas et al. 2020; Matrosova et al. 2023; Şeker 2024a; Teber et al. 2025). However, despite the taxonomic complexity within the *Nannospalax* populations in Türkiye, mitogenomic data remain scarce. Thus, generating complete mitochondrial genomes and reconstructing phylogenies are crucial for resolving species boundaries and elucidating evolutionary histories in this genus.

In this study, we aimed to characterize the complete mitochondrial genomes of two samples representing two species, *N. turcicus* (Homotypic synonym: *Nannospalax leucodon turcicus*) and *N. nehringi* , distributed in Türkiye, and to elucidate the phylogenetic relationships within the family Spalacidae using both newly generated mitogenomes and publicly available sequences from the GenBank database.

## 2 Materials and Methods

### 2.1 Sampling and gDNA Extraction

Two samples belonging to two taxa, *N. nehringi* and *N. turcicus*, were collected from Paşaçayırı, Kars (Northeast Anatolia) and Tekirdağ (Turkish Thrace) provinces of Türkiye, respectively. Field sampling and subsequent experimental procedures were conducted under official permits obtained separately for each species. Specimens of *N. turcicus* were collected with authorization from the General Directorate of Nature Conservation and National Parks, Ministry of Agriculture and Forestry of the Republic of Türkiye (Protocol No: E‐21264211‐288.04‐10,822,834). All laboratory procedures involving this species were approved by the Local Ethical Committee for Laboratory Animal Experimentation at Erciyes University (Protocol No: 23/061; April 5, 2023). Specimens of *N. nehringi* were obtained under permission No. 72784983‐488.04‐23381, issued by the Republic of Türkiye Ministry of Forestry and Water Affairs, Directorate of Nature Conservation and National Parks, in accordance with decision No. 2015/129. Ethical clearance for experimental procedures was granted by the Kafkas University Local Ethics Committee for Animal Experiments (No: 2015/129).

Various tissues from two samples were preserved in absolute ethanol and sent to the Department of Physiology, Faculty of Veterinary Medicine at Kafkas University, and the Department of Hunting and Wildlife at Artvin Çoruh University. Genomic DNA (gDNA) of the *Nannospalax* samples was extracted using the QIAGEN DNeasy Blood & Tissue Kit (QIAGEN, Germany), following the manufacturer's protocol. The concentration of gDNA was measured using the Qubit dsDNA BR Assay Kit with a Qubit 2.0 Fluorometer. To assess gDNA quality, 5 μL of each sample was run on a 1% agarose gel for 60 min and visualized under UV light. Successfully isolated gDNA samples were stored at −20°C until PCR experiments were conducted.

### 2.2 PCR Amplification of Mitochondrial Mitogenome

Complete mitochondrial genomes of two *Nannospalax* samples were obtained using the Long‐Range PCR method. The mitogenomes of the *Nannospalax* samples were amplified using two specific primer pairs: CrocAL1‐2024 L (forward 1): 5′‐GACCGTGCAAAGGTAGCATAATC−3′, CrocBH1‐13002H (reverse 1): 5′‐TAATTAAAAGGGCTCAGGCGTTG‐3′, ScVu‐11,712 L (forward 2): 5′‐AGAAGTAATCCATTGGTCTTAGGA‐3′, LuLu‐2503H (reverse 2): 5′‐CTCAGATCACGTAGGACTTTAATC‐3′ (reference). The Long‐Range PCR reactions were carried out using NEB LongAmp Taq 2X Master Mix (M0287S, New England Biolabs) and specific primer sets. Each PCR reaction mixture consisted of 12.5 μL of 1X NEB LongAmp Taq 2X Master Mix, 1.5 μL (0.6 μM) of each primer, and 1 μL of gDNA template (~30 ng). PCR amplifications were performed under the specified cycling conditions.

The PCR amplifications yielded two overlapping fragments, approximately 11 kb and 7.1 kb in length. The resulting amplicons were verified by electrophoresis on a 1% agarose gel, and their concentrations were determined using the Qubit dsDNA BR Assay Kit. PCR product concentrations were then standardized to 1 ng per 5 μL for sequencing library preparation. A negative control without template gDNA was included in all PCR experiments to check for contamination.

### 2.3 Library Preparation and Sequencing

Library preparation was performed using the Nextera XT DNA Library Prep Kit and the Nextera XT DNA Library Preparation Index Kit v2 Set A (Cat. No: FC‐131‐2001, Illumina), following the manufacturer's instructions. Library quantification was performed using the Qubit dsDNA HS Kit, and normalization was carried out using a bead‐based method to ensure equal representation of each library prior to sequencing. Sequencing was performed on the Illumina MiSeq platform (Illumina, San Diego, USA) at the Genome and Stem Cell Center (GenKok), Erciyes University, using the MiSeq Reagent Kit v2 (500 cycles).

### 2.4 Mitogenome Assembly and Annotation

Raw sequence reads for each mitogenome were imported as separate files into Geneious 9.1.8 software (Kearse et al. 2012) for further analysis. The raw data were trimmed and filtered using the BBDuk Trimming Tool in Geneious to remove adapters, reads shorter than 50 bp, uncertain (N) bases, and bases with Q‐scores below 25. Trimmed and filtered reads were assembled using Geneious’ Map to Reference tool (Highest Sensitivity/Slow), with the *N. ehrenbergi* mitogenome sequence (Accession number: NC_005315/AJ416891) as the reference. Trimmed and filtered contigs were also *de novo* assembled using Geneious software and subsequently remapped to the *N. ehrenbergi* mitogenome. Gene borders were examined and annotated using the MITOS2 Web Server (Donath et al. 2019). The circular mitochondrial genome was visualized using the CGView Server to generate a detailed graphical map (Grant and Stothard 2008). AT and GC skew values were calculated using the formulas AT skew = [A − T]/[A + T] and GC skew = [G − C] / [G + C], respectively, to assess nucleotide usage biases. Codon usage, relative synonymous codon usage (RSCU), and nucleotide composition were analyzed using MEGA11 software (Kumar et al. 2018). The RSCU matrix was visualized using Matrix2png (Pavlidis and Noble 2003), and a hierarchical cluster dendrogram of RSCUs was constructed using the single linkage Euclidean distance method. The Tandem Repeat Finder Web Server was used to identify repeat motifs in the *D‐loop* region (Benson 1999). The secondary structures of tRNAs were determined using the MITOS2 Web Server (Donath et al. 2019) and visualized with VARNA 3.93 software (Darty et al. 2009).

### 2.5 Phylogenetic and Genetic Distance Analyses

Phylogenetic analysis was carried out using complete mitochondrial genomes excluding the *D‐loop* region, derived from members of three subfamilies—Spalacinae, Myospalacinae, and Rhizomyinae—sourced from the GenBank database and from two *Nannospalax* individuals analyzed in the present study (Table S1). All sequences were combined and aligned using the MAFFT multiple sequence alignment program (Katoh et al. 2002). Gblocks (Talavera and Castresana 2007) was used to remove unreliably aligned regions from the mitogenome dataset.

In phylogenetic analysis, the GTR + G + I substitution model was selected as the best‐fit model for the mitogenome dataset, based on the Akaike Information Criterion (AIC) and Bayesian Information Criterion (BIC), using jModelTest v2.1.10 (Darriba et al. 2012). Phylogenetic trees were constructed using both Maximum Likelihood (ML) and Bayesian Inference (BI) methods. ML analysis was performed with 1000 bootstrap replications using MEGA11 (Tamura et al. 2021). BI analysis was used to construct the phylogenetic tree using MrBayes v3.2.6 (Ronquist et al. 2012) and conducted with a Markov Chain Monte Carlo (MCMC) approach, employing four chains (three heated and one cold). The MCMC sampler was run for 3,000,000 generations, with trees sampled every 1000 generations. The first 25% of samples were discarded as burn‐in. This number of generations was sufficient to reduce the standard deviation of split frequencies to below 0.01, indicating convergence. The remaining posterior samples were used to construct a Bayesian consensus tree. The potential scale reduction factor (PSRF), which approached 1.0 for all parameters, was used to assess the adequacy of the MCMC run. Tracer v1.7 (Rambaut et al. 2018) was used to assess convergence to stationarity via trace plots and to verify the effective sample sizes (ESS > 200) of model parameters. The phylogenetic tree resulting from the BI analysis was visualized using FigTree v1.4.4 (Rambaut 2009). The mitogenome of *Meriones unguiculatus* from the family Muridae (Accession number: NC_023263/KF425526, Li, Lu, and Wang 2016; Li, Shi, et al. 2016) was used as outgroup in the phylogenetic analysis.

Complete mitochondrial genomes and *cytochrome b* (*CYTB*) sequences of the superspecies *N. nehringi* and *N. turcicus* from Türkiye were analyzed to estimate pairwise genetic distances using the Kimura 2‐Parameter (K2P) model, with 10,000 bootstrap replicates performed in MEGA11 (Tamura et al. 2021).

## 3 Results

### 3.1 General Characteristics of Mitogenomes

The complete mitochondrial genomes of two BMR specimens, representing *N. nehringi* *and N. turcicus* from Türkiye, were sequenced at an average coverage of approximately 2885× and 1875× respectively. The assembled genome lengths were 16,424 bp for specimen of *N. nehringi* and 16,421 bp for specimen of *N. turcicus*. Evaluation of results obtained from BLAST analysis in conjunction with geographical sampling data indicated that the two BMR mitogenomes belonged to *N. turcicus* (collected from Tekirdağ in Turkish Thrace), within the *N. leucodon* superspecies, and *N. nehringi* (collected from Kars in Northeast Anatolia), within the *N. nehringi* superspecies. In the present study, the taxonomic classification of the examined *Nannospalax* specimens follows the taxonomic nomenclature proposed by Kankılıç, Çelikbilek, et al. (2024), Kankılıç, Civelek, and Köse (2024), and Németh et al. (2024).

**FIGURE 1:**
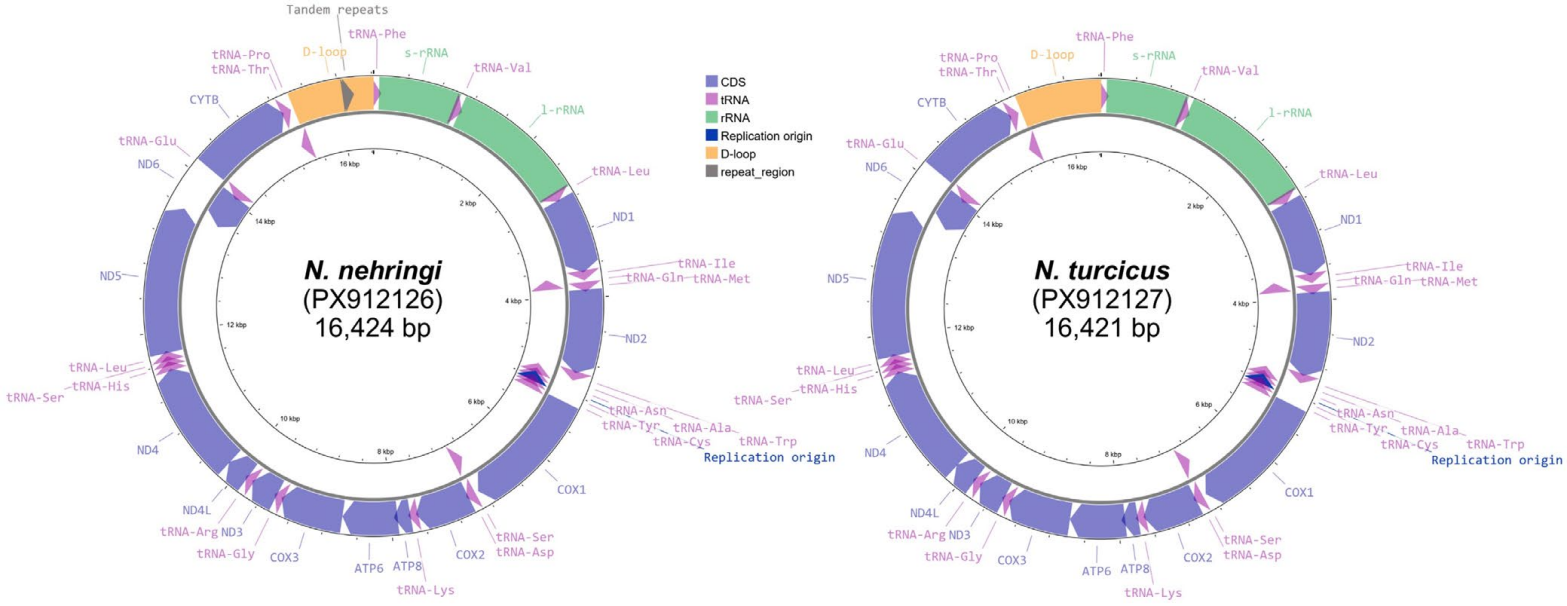
Circular maps of the complete mitochondrial genomes of *N. nehringi* (PX912126) and *N. turcicus* (PX912127), reconstructed from specimens collected in Türkiye. *CDS*, *tRNAs*, *rRNAs*, the replication origin, *D‐loop* region and tandem repeat regions are indicated using different color codes.

Regarding the general characteristics of mitogenomes, the complete mitogenome of the Turkish BMR specimens was annotated and characterized in detail (Figure 1, Table 1). Figure 1 presented the circular map of the mitochondrial genomes (mitogenome) obtained from *Nannospalax* specimens collected in Türkiye. The mitogenome of Turkish *N. nehringi* and *N. turcicus* exhibited the typical vertebrate mitochondrial gene composition, including 13 protein‐coding genes (*PCGs*), 22 transfer RNA genes (*tRNAs*), two ribosomal RNA genes (*12S* and *16S rRNAs*), a replication origin (O_*L*_), and a control region (*D‐loop*). The O_*L*_, *ND6*, *tRNA* ^*Asn*^, *tRNA* ^*Gln*^, *tRNA* ^*Ala*^, *tRNA* ^*Cys*^, *tRNA* ^*Tyr*^, *tRNA* ^*Ser2*^, *tRNA* ^*Glu*^, and *tRNA* ^*Pro*^ were encoded on the light strand (L‐strand), whereas the two *rRNAs*, 12 *PCGs*, and 14 *tRNAs* were encoded on the heavy strand (H‐strand) (Figure 1, Table 1). The origin of light strand replication (O_*L*_) was located within the WANCY *tRNA* gene cluster. Both mitogenomes contained intergenic spacer regions as well as overlapping sequences. The largest intergenic spacer in both genomes was a 9 bp region located between *tRNA* ^*Leu2*^ and *ND1*. The longest overlapping region was identified between *ATP6* and *ATP8*, spanning 46 bp in *N. nehringi* and 43 bp in *N. turcicus*.

**TABLE 1:** Genomic organization and structural annotation of the mitochondrial genomes of *N. nehringi* and *N. turcicus*, derived from specimens collected in Türkiye.

Gene	Species	Direction	Position	Length (bp)	Intergenic spacer (bp)	Initiation codons	Stop codons	Anticodon	AT‐skew
*tRNA* ^*Phe*^	*N. nehringi* (PX912126)	H	1–69	69	0			GAA	0.300
	*N. turcicus* (PX912127)	H	1–68	68	0			GAA	0.300
*12S rRNA*	*N. nehringi* (PX912126)	H	70–1027	958	0				0.223
	*N. turcicus* (PX912127)	H	69–1026	958	0				0.225
*tRNA* ^*Val*^	*N. nehringi* (PX912126)	H	1028–1093	66	0			TAC	0.235
	*N. turcicus* (PX912127)	H	1027–1092	66	0			TAC	0.235
*16S rRNA*	*N. nehringi* (PX912126)	H	1094–2665	1572	0				0.235
	*N. turcicus* (PX912127)	H	1093–2665	1573	0				0.232
*tRNA* ^*Leu2*^	*N. nehringi* (PX912126)	H	2666–2740	75	+9			TAA	0.163
	*N. turcicus* (PX912127)	H	2666–2740	75	+9			TAA	0.143
*ND1*	*N. nehringi* (PX912126)	H	2750–3695	946	0	ATT	T‐‐		0.093
	*N. turcicus* (PX912127)	H	2750–3695	946	0	ATT	T‐‐		0.093
*tRNA* ^*Ile*^	*N. nehringi* (PX912126)	H	3696–3763	68	−3			GAT	0.111
	*N. turcicus* (PX912127)	H	3696–3763	68	−3			GAT	0.111
*tRNA* ^*Gln*^	*N. nehringi* (PX912126)	L	3761–3831	71	+5			TTG	0.116
	*N. turcicus* (PX912127)	L	3761–3831	71	+5			TTG	0.211
*tRNA* ^*Met*^	*N. nehringi* (PX912126)	H	3837–3905	69	0			CAT	0.135
	*N. turcicus* (PX912127)	H	3837–3905	69	0			CAT	0.167
*ND2*	*N. nehringi* (PX912126)	H	3906–4947	1042	0	ATT	T‐‐		0.092
	*N. turcicus* (PX912127)	H	3906–4947	1042	0	ATT	T‐‐		0.091
*tRNA* ^*Trp*^	*N. nehringi* (PX912126)	H	4948–5013	66	+3			TCA	0.179
	*N. turcicus* (PX912127)	H	4948–5014	67	+3			TCA	0.200
*tRNA* ^*Ala*^	*N. nehringi* (PX912126)	L	5017–5085	69	0			TGC	0.022
	*N. turcicus* (PX912127)	L	5018–5086	69	0			TGC	0.095
*tRNA* ^*Asn*^	*N. nehringi* (PX912126)	L	5086–5158	73	0			GTT	0.318
	*N. turcicus* (PX912127)	L	5087–5159	73	0			GTT	0.348
*O*_*L*_	*N. nehringi* (PX912126)	L	5159–5190	32	0				0.412
	*N. turcicus* (PX912127)	L	5160–5192	33	0				0.467
*tRNA* ^*Cys*^	*N. nehringi* (PX912126)	L	5191–5257	67	+1			GCA	0.059
	*N. turcicus* (PX912127)	L	5193–5259	67	+1			GCA	0.000
*tRNA* ^*Tyr*^	*N. nehringi* (PX912126)	L	5259–5322	64	+1			GTA	−0.135
	*N. turcicus* (PX912127)	L	5261–5323	63	+1			GTA	−0.158
*COX1*	*N. nehringi* (PX912126)	H	5324–6868	1545	−3	ATG	TAA		0.007
	*N. turcicus* (PX912127)	H	5325–6869	1545	−3	ATG	TAA		−0.009
*tRNA* ^*Ser2*^	*N. nehringi* (PX912126)	L	6866–6934	69	+3			TGA	0.136
	*N. turcicus* (PX912127)	L	6867–6935	69	+3			TGA	0.116
*tRNA* ^*Asp*^	*N. nehringi* (PX912126)	H	6938–7005	68	0			GTC	0.115
	*N. turcicus* (PX912127)	H	6939–7006	68	0			GTC	0.120
*COX2*	*N. nehringi* (PX912126)	H	7006–7689	684	+1	ATG	TAA		0.126
	*N. turcicus* (PX912127)	H	7007–7690	684	+1	ATG	TAA		0.122
*tRNA* ^*Lys*^	*N. nehringi* (PX912126)	H	7691–7755	65	+1			TTT	0.128
	*N. turcicus* (PX912127)	H	7692–7754	63	+1			TTT	0.128
*ATP8*	*N. nehringi* (PX912126)	H	7757–7963	207	−46	ATG	TAG		0.217
	*N. turcicus* (PX912127)	H	7756–7959	204	−43	ATG	TAA		0.232
*ATP6*	*N. nehringi* (PX912126)	H	7918–8598	681	−1	ATG	TAA		0.066
	*N. turcicus* (PX912127)	H	7917–8597	681	−1	ATG	TAA		0.089
*COX3*	*N. nehringi* (PX912126)	H	8598–9381	784	0	ATG	T‐‐		0.048
	*N. turcicus* (PX912127)	H	8597–9380	784	0	ATG	T‐‐		0.033
*tRNA* ^*Gly*^	*N. nehringi* (PX912126)	H	9382–9449	68	0			TCC	0.156
	*N. turcicus* (PX912127)	H	9381–9447	67	0			TCC	0.200
*ND3*	*N. nehringi* (PX912126)	H	9450–9797	348	+3	ATA	TAA		0.033
	*N. turcicus* (PX912127)	H	9448–9795	348	+3	ATA	TAA		0.032
*tRNA* ^*Arg*^	*N. nehringi* (PX912126)	H	9801–9869	69	0			TCG	0.120
	*N. turcicus* (PX912127)	H	9799–9867	69	0			TCG	0.120
*ND4L*	*N. nehringi* (PX912126)	H	9870–10,166	297	−7	ATA	TAA		−0.063
	*N. turcicus* (PX912127)	H	9868–10,164	297	−7	ATA	TAA		−0.015
*ND4*	*N. nehringi* (PX912126)	H	10,160–11,536	1377	+1	ATG	TAA		0.085
	*N. turcicus* (PX912127)	H	10,158–11,534	1377	+1	ATG	TAA		0.080
*tRNA* ^*His*^	*N. nehringi* (PX912126)	H	11,538–11,604	67	0			GTG	0.154
	*N. turcicus* (PX912127)	H	11,536–11,602	67	0			GTG	0.154
*tRNA* ^*Ser1*^	*N. nehringi* (PX912126)	H	11,605–11,663	59	0			GCT	0.125
	*N. turcicus* (PX912127)	H	11,603–11,662	59	0			GCT	0.125
*tRNA* ^*Leu1*^	*N. nehringi* (PX912126)	H	11,664–11,733	70	0			TAG	0.191
	*N. turcicus* (PX912127)	H	11,662–11,731	70	0			TAG	0.174
*ND5*	*N. nehringi* (PX912126)	H	11,734–13,560	1827	−17	ATT	TAA		0.148
	*N. turcicus* (PX912127)	H	11,732–13,558	1827	−17	ATT	TAA		0.127
*ND6*	*N. nehringi* (PX912126)	L	13,544–14,068	525	0	ATG	AGG		0.415
	*N. turcicus* (PX912127)	L	13,542–14,066	525	0	ATG	AGA		0.366
*tRNA* ^*Glu*^	*N. nehringi* (PX912126)	L	14,069–14,137	69	+4			TTC	0.234
	*N. turcicus* (PX912127)	L	14,067–14,135	69	+4			TTC	0.234
*CYTB*	*N. nehringi* (PX912126)	H	14,142–15,281	1140	+4	ATG	TAA		0.054
	*N. turcicus* (PX912127)	H	14,140–15,279	1140	+4	ATG	TAA		0.046
*tRNA* ^*Thr*^	*N. nehringi* (PX912126)	H	15,286–15,354	69	0			TGT	0.234
	*N. turcicus* (PX912127)	H	15,284–15,351	68	0			TGT	0.200
*tRNA* ^*Pro*^	*N. nehringi* (PX912126)	L	15,355–15,419	65	0			TGG	0.150
	*N. turcicus* (PX912127)	L	15,352–15,417	66	0			TGG	0.268
*D‐loop*	*N. nehringi* (PX912126)	H	15,420–16,424	1005	0				−0.008
	*N. turcicus* (PX912127)	H	15,418–16,421	1004	0				−0.031

*Note:* The table lists the gene order and genomic features, including strand direction (H, heavy strand; L, light strand), genomic positions, gene lengths, intergenic spacer sizes, initiation and stop codons for *PCGs*, *tRNA* anticodons, and AT‐skew values.

**FIGURE 2:**
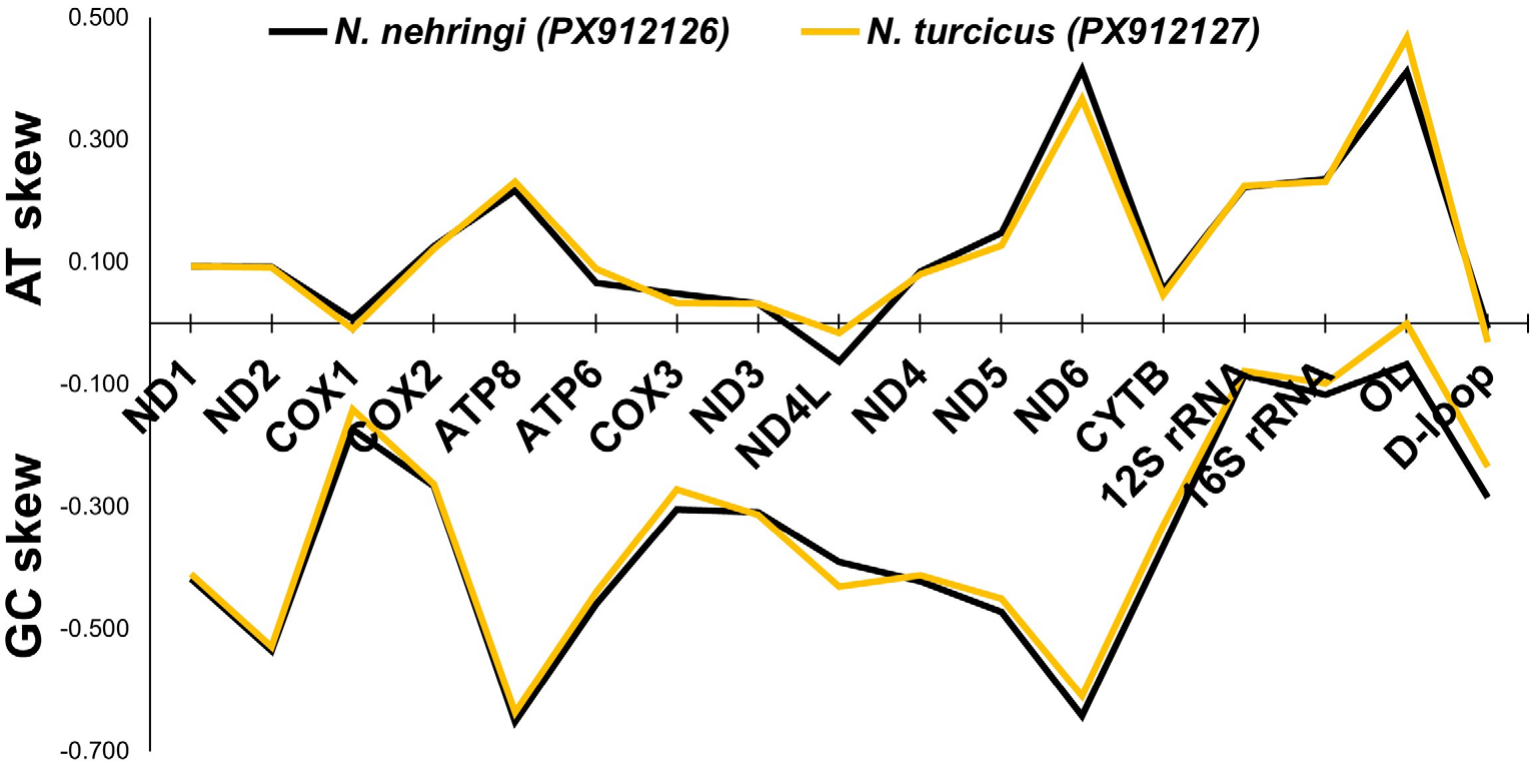
Grap illustrating AT and GC skews of genes in the mitochondrial genomes of *N. nehringi* and *N. turcicus*. Positive and negative skew values indicate nucleotide compositional bias among *PCGs*, *rRNAs*, *tRNAs*, the *D‐loop* region and the O_*L*_. Differences in skew patterns among genomic regions are shown comparatively between the two mitogenomes.

In terms of nucleotide composition, the mitogenome of *N. nehringi* exhibited 34.70% Adenine (A), 27.59% Thymine (T), 24.92% Cytosine (C), and 12.79% Guanine (G), whereas the mitogenome of *N. turcicus* comprised 34.52% A, 27.79% T, 24.54% C, and 13.15% G (Table 2). In both species, the nucleotide composition followed the order A > T > C > G. Both Turkish *Nannospalax* mitogenomes exhibited a positive AT‐skew and a negative GC‐skew. Region‐specific analysis revealed that the highest GC‐skew values were observed in the *rRNA* genes and the origin of light‐strand replication (O_*L*_), whereas the lowest values occurred in *ATP8* and *ND6* genes (Figure 2).

**TABLE 2:** Nucleotide composition of *PCGs*, *rRNA* and *tRNA* genes, and the *D‐loop* region in mitochondrial genomes of *N. nehringi* and *N. turcicus* derived from specimens collected in Türkiye.

	Species	Size (bp)	A (%)	C (%)	G (%)	T (%)	A + T (%)	G + C (%)	AT Skew	GC Skew
Whole genome	*N. nehringi* (PX912126)	16,424	34.70	24.92	12.79	27.59	62.29	37.71	0.114	−0.322
	*N. turcicus* (PX912127)	16,421	34.52	24.54	13.15	27.79	62.31	37.69	0.108	−0.302
*PCGs*	*N. nehringi* (PX912126)	11,403	34.02	26.32	11.48	28.19	62.20	37.80	0.094	−0.393
	*N. turcicus* (PX912127)	11,400	33.82	25.93	11.81	28.44	62.26	37.74	0.087	−0.374
*rRNA* genes	*N. nehringi* (PX912126)	2530	38.02	21.11	17.11	23.75	61.78	38.22	0.231	−0.104
	*N. turcicus* (PX912127)	2531	38.13	20.70	17.27	23.90	62.03	37.97	0.229	−0.091
*tRNA* genes	*N. nehringi* (PX912126)	1495	35.99	22.07	15.32	26.62	62.61	37.39	0.150	−0.181
	*N. turcicus* (PX912127)	1491	36.02	22.13	15.83	26.02	62.04	37.96	0.161	−0.166
*D‐loop*	*N. nehringi* (PX912126)	1005	31.94	22.89	12.74	32.44	64.38	35.62	−0.008	−0.285
	*N. turcicus* (PX912127)	1004	31.18	22.01	13.65	33.17	64.34	35.66	−0.031	−0.235

*Note:* The table presents the nucleotide frequencies (A, C, G, and T), genome size, A + T and G + C contents, and AT‐ and GC‐skew values calculated for the whole mitochondrial genome and its major genomic regions.

### 3.2 *PCGs* and Codon Usage

**FIGURE 3:**
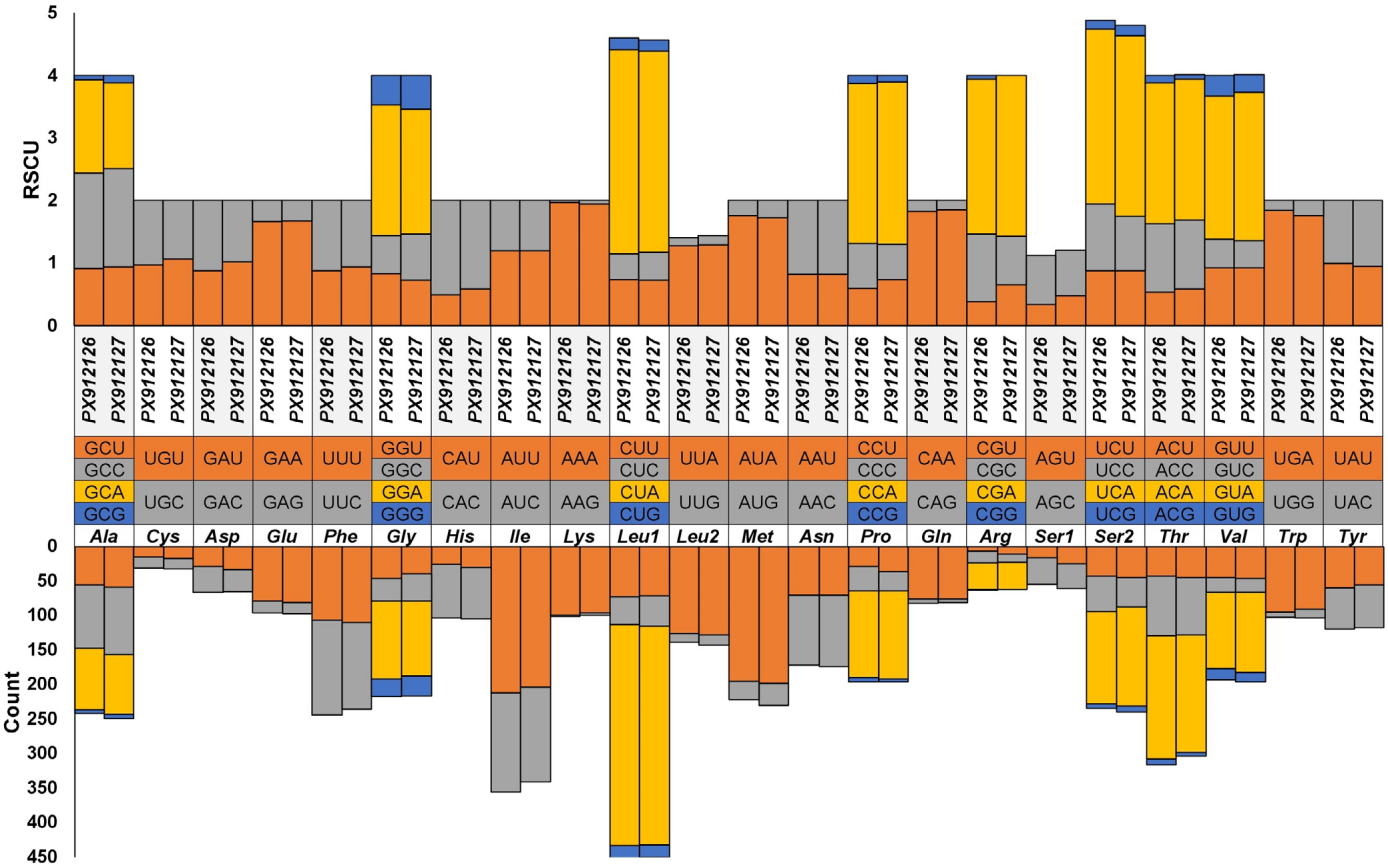
RSCU values and codon frequency distributions of mitochondrial *PCGs* in *N. nehringi* (PX912126) and *N. turcicus* (PX912127). The upper panel shows RSCU values for synonymous codons of each amino acid, whereas the lower panel presents the corresponding codon counts. Differences in codon usage bias and nucleotide composition patterns between the two mitogenomes are illustrated comparatively.

The Relative Synonymous Codon Usage (RSCU) values and codon frequencies for *N. nehringi* and *N. turcicus* were presented in Figure 3. Among all amino acids, Leucine1 (451 and 450 codons, respectively), Isoleucine (355 and 340), and Threonine (316 and 303) were the most frequently encoded. In contrast, Cysteine (31 and 32 codons), Serine1 (54 and 60), and Arginine (63 and 62) showed the lowest codon usage. Codon usage analysis revealed that *CUA* (Leucine1), *AUC* (Isoleucine), and *AUA* (Methionine) were the most commonly used codons, whereas *CGG* (Arginine), *AAG* (Lysine), and *CCG* (Proline) were among the least utilized codons. *ATG* was identified as the predominant initiation codon in the *PCGs* of both *N. nehringi* and *N. turcicus*, accounting for approximately 61.5% of start codons, while *ATA* was the least frequently utilized (15.4%). Termination of mitochondrial PCGs was achieved using standard stop codons *TAA*, *TAG*, *AGG*, or *AGA*; however, *ND1*, *ND2*, and *COX3* genes terminated with incomplete stop codons (*T‐‐*).

**FIGURE 4:**
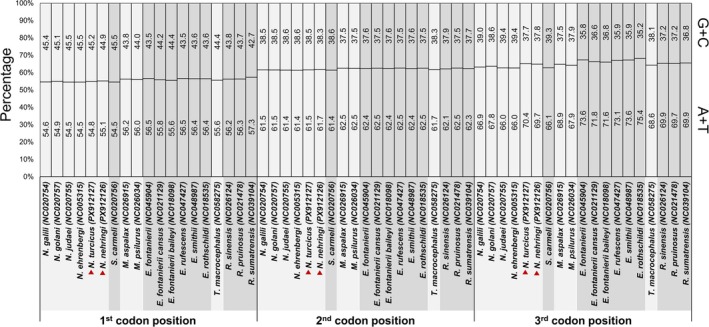
Nucleotide composition at the 1st, 2nd and 3rd codon positions of mitochondrial *PCGs* in members of the family *Spalacidae*, including *N. nehringi* and *N. turcicus*. Stacked bars represent the relative proportions of A + T and G + C contents at each codon position.

**FIGURE 5:**
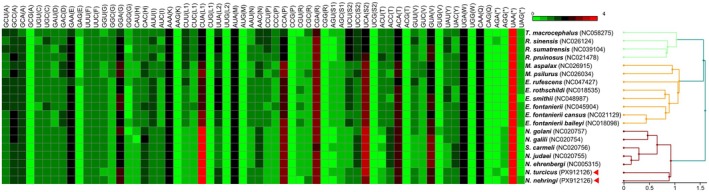
Heatmap of RSCU frequencies in species of the family *Spalacidae*. The clustered dendrogram was generated using the single‐linkage method based on Euclidean distance. Color intensity represents RSCU frequency values, ranging from low usage frequency (green) to high usage frequency (red).

**FIGURE 6:**
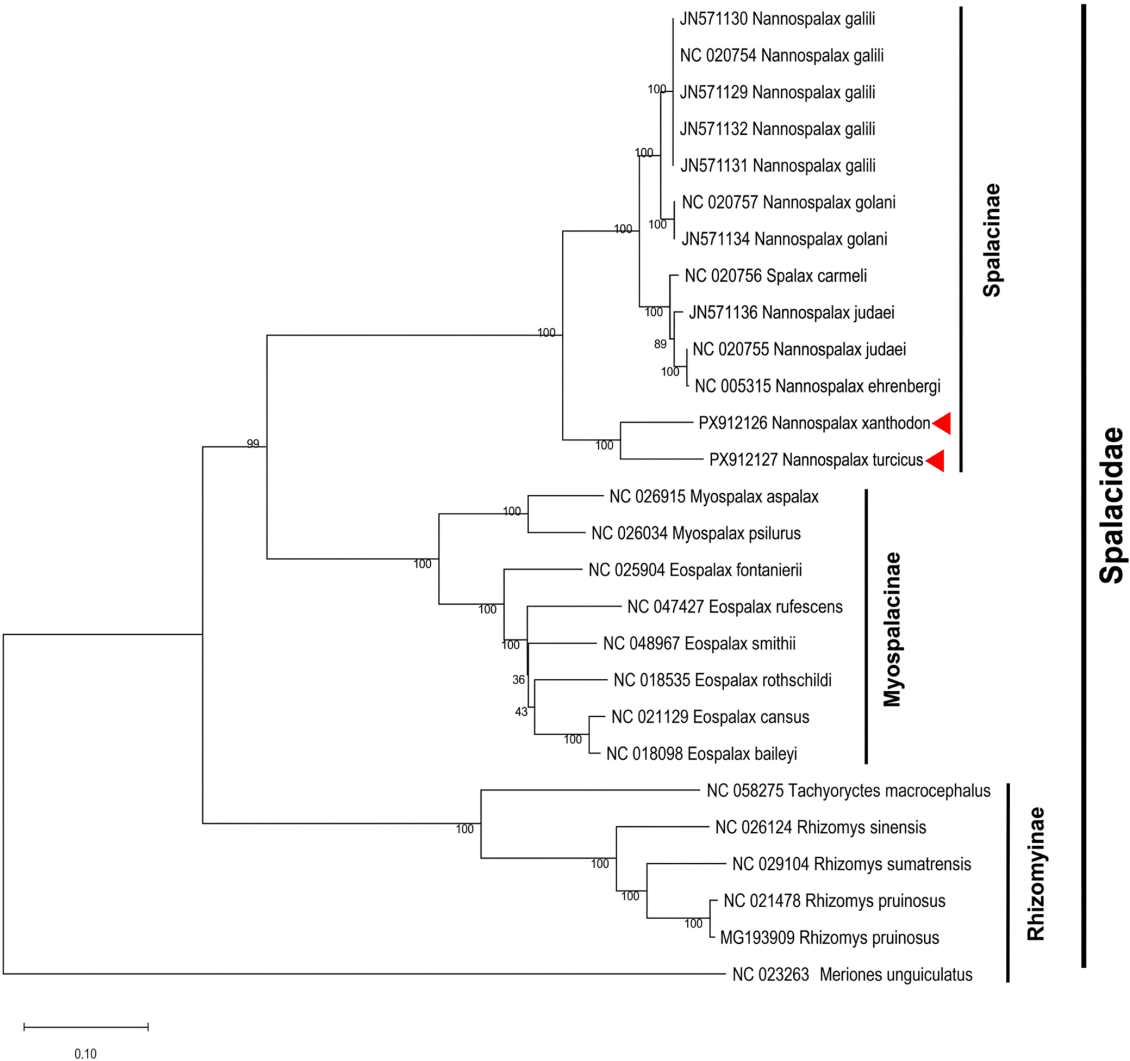
Maximum Likelihood phylogenetic tree based on complete mitochondrial genomes excluding the D‐loop region, constructed using the GTR + G + I substitution model with 1000 bootstrap replicates, illustrating the phylogenetic relationships within the family *Spalacidae*.

**FIGURE 7:**
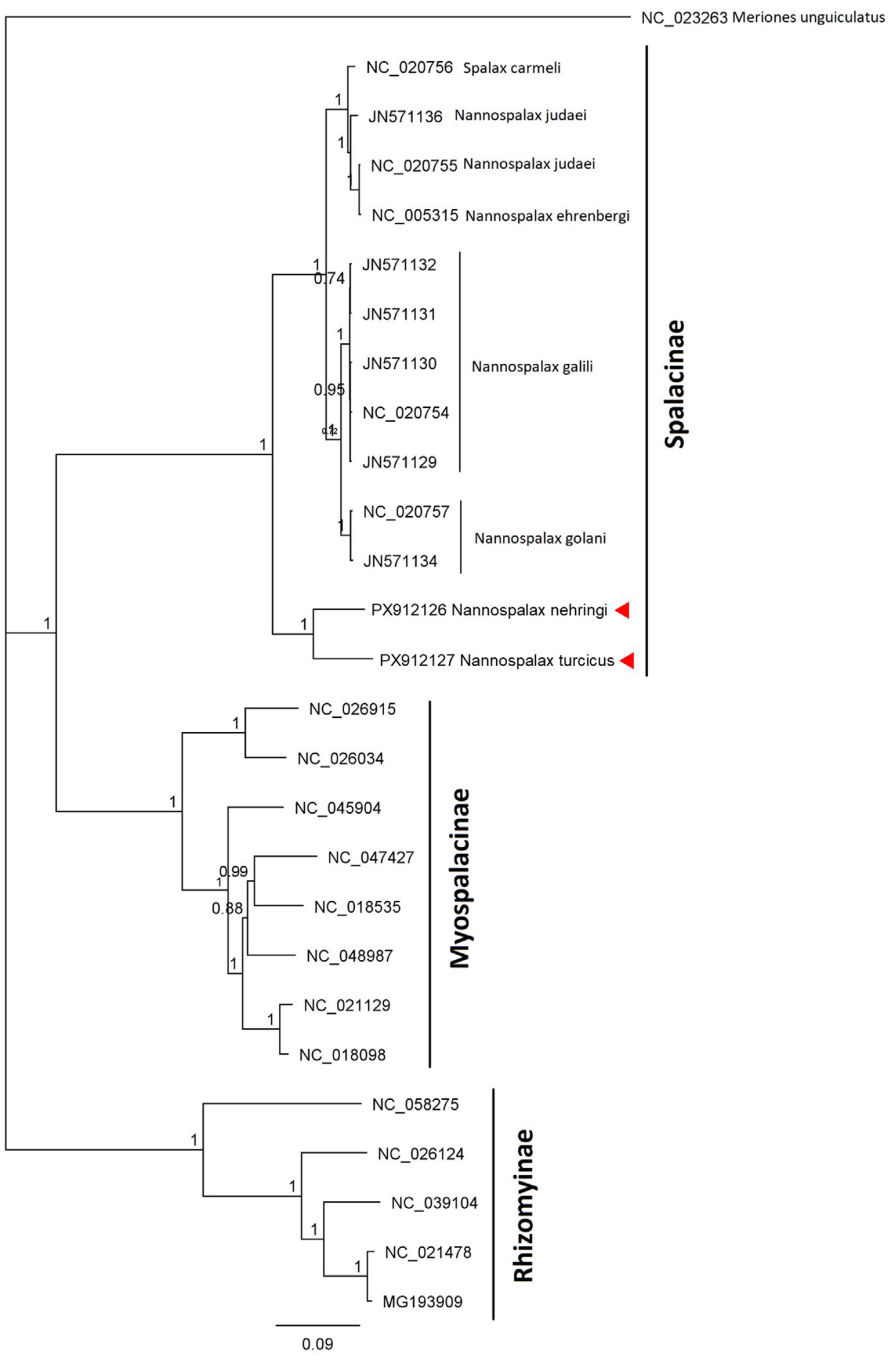
Bayesian Inference phylogenetic tree based on complete mitochondrial genomes excluding the D‐loop region, generated with the GTR + G + I substitution model over 3,000,000 generations, showing the phylogenetic relationships within the family *Spalacidae*.

Nucleotide composition analysis at the first, second, and third codon positions (A + T and G + C content) showed no interspecific variation at the first and second positions. However, the third codon position exhibited species‐specific variation (Figure 4). Among the genera examined, *Nannospalax* showed the lowest A + T content at the third codon position, whereas *Eospalax* exhibited the highest. Notably, *N. nehringi* and *N. turcicus* displayed higher A + T content at the third codon position compared to other *Nannospalax* species. Based on RSCU data, species were clustered using single‐linkage hierarchical clustering (Figure 5), and the resulting pattern mirrored the tree topologies obtained through Maximum Likelihood (ML) and Bayesian Inference (BI) analyses (Figures 6 and 7).

### 3.3 Ribosomal RNA ( *rRNA* ) and Transfer RNA ( *tRNA* ) Genes

Regarding *RNA* genes, the 12S *rRNA* gene was 958 bp in length in both mitogenomes, while the 16S *rRNA* gene measured 1572 bp in *N. nehringi* and 1573 bp in *N. turcicus* (Table 1). The 22 *tRNA* genes ranged from 59 bp to 75 bp (Table 1). All *tRNAs* displayed typical cloverleaf secondary structures, with the exception of *tRNA* ^*Ser1*^ (*GCT*), which lacked the DHU arm. Structural comparisons revealed slight differences in tRNA secondary structures, particularly in the (variable loop) V‐loop regions of *tRNA* ^*Asn*^ and *tRNA* ^*Thr*^, and variations in the TΨC‐loop were detected in *tRNA* ^*Gly*^, *tRNA* ^*Lys*^, *tRNA* ^*Phe*^, and *tRNA* ^*Pro*^. DHU arm variation was observed exclusively in the *tRNA* ^*Asp*^ gene (Figures S1 and S2).

### 3.4 The Origin of Replication for the Light Strand (OL) and the Control Region (D‐Loop)

**FIGURE 8:**
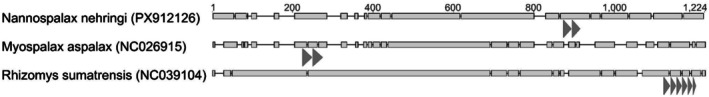
Tandem repeats in the *D‐loop* regions of the mitochondrial genomes of *N. nehringi*, *M. aspalax* , and *R. sumatrensis* . No tandem repeats were detected in the D‐loop region of *N. turcicus*.

The O_*L*_ region was 32 bp in *N. nehringi* and 33 bp in *N. turcicus* (Table 1), including the conserved 5′‐CTTCT‐3′ motif. The *D‐loop* region was 1004 bp in *N. nehringi* and 1005 bp in *N. turcicu*s (Table 1). While no tandem repeats were identified in the *D‐loop* of *N. turcicus*, a 22‐bp repeat motif repeated approximately 2.2 times was detected in *N. nehringi* (Figure 8).

### 3.5 Phylogenetic Relationships of Taxa Within the Family *Spalacidae*

Phylogenetic analyses based on complete mitogenomes using both ML and BI methods produced similar tree topologies (Figures 6 and 7). The mitogenomes of *N. turcicus* and *N. nehringi* were distinct from other species of the genus *Nannospalax* registered as *N. galili*, *N. golani*, *N. carmeli*, *N. judaei*, and *N. ehrenbergi* , and together formed a monophyletic group (Figures 6 and 7). Mean genetic distance between *N. nehringi* and *N. turcicus* was 0.0926 (9.26%) based on mitogenomic data, whereas it was 0.1177 (11.77%) based on *CYTB* data. Comparison with previously published data showed that the mean genetic divergence between the Tekirdağ BMR specimen (*N. turcicus*) and two conspecific sequences (OR751035 and OR751036) was low (0.75%), whereas its distance from two BMR sequences from Eceabat‐Çanakkale ( *N. leucodon* ; MH300078 and MH300079) was substantially higher (4.6%). Additionally, genetic divergence between the *N. nehringi* *CYTB* sequence from Paşaçayırı, Kars and the sequence from Sarıkamış, Kars (JX451854) was 1.96%.

## 4 Discussion

The mitochondrial divergence observed between *N. turcicus* and *N. leucodon* specimens may reflect genetic differentiation among currently recognized lineages within the Turkish Thrace region. The genus *Nannospalax*, which includes BMRs, constitutes one of the most notable examples of ongoing systematic differentiation among the mammalian fauna of Türkiye (Kankılıç, Civelek, and Köse 2024; Kankılıç, Çelikbilek, et al. 2024; Németh et al. 2024). A comparative analysis of the complete mitochondrial genomes of *Spalacidae* species available in GenBank revealed notable differences in sequence length (Table S1). These sequence length variations may reflect evolutionary divergence within the family and potential species‐specific mitochondrial adaptations. Furthermore, the location of the origin of O_*L*_ within the WANCY *tRNA* gene cluster is consistent with previous reports (Gadaleta et al. 1989; Seutin et al. 1994). As observed in mitogenomes of various mammalian lineages, including jerboas (*Scarturus williamsi*; İbiş 2020), voles ( *Prometheomys schaposchnikowi* (İbiş et al. 2020), *Chionomys roberti* and *C. nivalis* ; Şeker et al. 2022), moles (*Talpa martinorum*; Demirtaş et al. 2022), squirrels ( *Sciurus anomalus* and *S. vulgaris* ; İbiş et al. 2022), shrews ( *Crocidura leucodon* , *C. gueldenstaedtii* and *C. mimuli* ; İbiş et al. 2023), hedgehogs ( *Erinaceus concolor* ; Şeker 2024b), dormice ( *Dryomys laniger* , *D. nitedula* and *Glis glis* ; İbiş et al. 2025), and ground squirrels ( *Spermophilus citellus* , *S. taurensis* and *S. xanthopyrmnus*; İbiş et al. 2025), the presence of intergenic spacer regions and overlapping sequences appears to be a shared genomic feature.

The observed compositional bias (A > T > C > G) was consistent with patterns reported in other mammalian mitochondrial genomes such as the Argali sheep *Ovis ammon* (Wang et al. 2019), *S. williamsi* (İbiş 2020), *P. schaposchnikowi* (İbiş et al. 2020), *Bos taurus* (Arbizu et al. 2022), *S. anomalus* and *S. vulgaris* (İbiş et al. 2022), *C. roberti* and *C. nivalis* (Şeker et al. 2022), *Tapirus pinchaque* (Gutiérrez et al. 2023), *D. laniger* , *D. nitedula* , *G. glis* , *S. citellus* , *S. taurensis* , and *S. xanthopyrmnus* (İbiş et al. 2025), and *Capra aegagrus* and *C. hircus* (Teber et al. 2025).

Codon usage patterns also provide significant insights. The prevalence of *ATG* as a start codon has also been reported in mitochondrial *PCGs* of other species within the family *Spalacidae* (Cai et al. 2020, 2019; Li, Lu, and Wang 2016; Su et al. 2013; Yuan et al. 2016). Moreover, the occurrence of incomplete stop codons (*T‐‐*) is a common feature in metazoan mitogenomes (J. L. Boore 2004; Chen et al. 2022; Song, Chen, et al. 2020; Song, Gao, et al. 2020; Yang et al. 2020; Yue et al. 2015) and has also been previously documented in other *Spalacidae* species (Cai et al. 2020, 2019; Li, Lu, and Wang 2016; Su et al. 2013). The limited variation in A + T/G + C composition at the third codon position within each genus is consistent with its recognized role in shaping codon usage bias and synonymous substitution patterns (Kosiol et al. 2007). Variation in codon usage bias among species has been shown to correlate closely with their phylogenetic relationships (Xiao et al. 2022), and previous studies have demonstrated that phylogenetic analyses based on codon usage patterns can provide valuable insights into evolutionary history (Roychoudhury et al. 2011; Wu et al. 2023). This concordance supports the reliability of RSCU‐based clustering in reflecting phylogenetic relationships within *Spalacidae*.

Regarding RNA structure, the lack of a DHU arm in *tRNA* ^*Ser1*^ (*GCT*) is a known characteristic of metazoan mitochondrial *tRNAs* (Garey and Wolstenholme 1989). Furthermore, the TΨC‐loop variations observed in several *tRNAs* are significant, as this loop plays a critical role in *tRNA* modifications necessary for proper aminoacylation and correct positioning within the ribosomal A site (Roovers et al. 2021).

The non‐coding regions, specifically the O_*L*_ and *D‐loop*, play essential roles in initiating replication in opposite directions (Brown et al. 2005). The O_*L*_ location is a conserved feature of vertebrate mitogenomes (Seutin et al. 1994). The *D‐loop* is also known to harbor tandem repeats that can offer insights into evolutionary relationships (Xie et al. 2006). Tandem repeats within the *D‐loop* region were identified in only three species, *Myospalax aspalax* (Yuan et al. 2016), *Rhizomys sumatrensis* (Xu et al. 2018), and *N. nehringi* among the mitogenomes analyzed. Notably, this study reported for the first time the presence of tandem repeats in the *D‐loop* region of *N. nehringi* within the family *Spalacidae*.

From a broader phylogenetic perspective, the genus *Nannospalax* represents a striking example of ongoing taxonomic diversification, likely driven by geographic isolation and ecological differentiation (Kankılıç, Civelek, and Köse 2024; Kankılıç, Çelikbilek, et al. 2024; Németh et al. 2024; Kankılıç et al. 2026). Previous studies (Cai et al. 2019; Yuan et al. 2016) have shown that *Spalacidae* taxa are divided into three subfamilies: *Myospalacinae*, *Spalacinae*, and *Rhizomyinae*. Using concatenated sequences, Németh et al. (2024) indicated that the *Nannospalax* population in Turkish Thrace represents a distinct species, *N. turcicus*, which was subsequently included in the updated checklist by (Sözen and Çolak 2025). Moreover, Kankılıç, Civelek, and Köse (2024) revealed that Anatolian *Nannospalax* specimens, excluding southeastern *N. ehrenbergi* , clustered into lineages corresponding to *N. xanthodon*, *N. nehringi* , *N. tuncelicus*, and *N. cilicicus*. Specimens from Kars were genetically assigned to *N. nehringi* (Kankılıç et al. 2026). However, Sözen and Çolak (2025) noted that *N. munzuri* samples had not been included in phylogenetic analyses and did not consider *N. nehringi* a valid taxon, emphasizing that northeastern populations should be classified under *N. xanthodon*.

The clear mitochondrial divergence observed between *N. turcicus* and *N. leucodon* specimens indicates possible inter‐population differentiation within the Turkish Thrace region. High levels of genetic variation between and within the constituent species of the BMR superspecies, *N. nehringi* and *N. leucodon* , have been consistently reported (Bugarski‐Stanojević et al. 2022; Kankılıç, Civelek, and Köse 2024; Kankılıç, Çelikbilek, et al. 2024; Kryštufek et al. 2012; Németh et al. 2024; Matur et al. 2019), supporting the notion of considerable intraspecific genetic structuring. Extensive chromosomal rearrangements in BMRs have led to the formation of numerous lineages with altered karyotypes, which can cause meiotic difficulties and result in reproductive isolation (Bugarski‐Stanojević et al. 2022). The taxonomy remains incomplete due to the absence of a comprehensive molecular evaluation based on a large dataset (Carleton and Musser 2005), and unique adaptations for subterranean living have shaped their phenotype, complicating the understanding of true phylogenetic relationships (Savić et al. 2017).

In conclusion, although based on a limited number of specimens, these analyses revealed substantial genetic diversity within and between *N. nehringi* and *N. turcicus*. The observed high intra‐ and interspecific divergence may reflect historical biogeographic events and suggest the presence of cryptic lineages. To better resolve taxonomic boundaries, further studies incorporating broader sampling and multilocus datasets are essential.

## Author Contributions

**Barış Yıldız:** conceptualization (equal), data curation (equal), formal analysis (equal), investigation (equal), software (equal), visualization (equal), writing – original draft (equal), writing – review and editing (equal). **Ahmet Yesari Selçuk:** conceptualization (equal), data curation (equal), formal analysis (equal), investigation (equal), software (equal), validation (equal), visualization (equal), writing – original draft (equal), writing – review and editing (equal). **Saffet Teber:** conceptualization (equal), data curation (equal), formal analysis (equal), investigation (equal), software (equal), validation (equal), visualization (equal). **Mehmet Baran:** conceptualization (equal), data curation (equal), formal analysis (equal), investigation (equal), software (equal), validation (equal), visualization (equal). **Yüksel Coşkun:** conceptualization (equal), formal analysis (equal), investigation (equal), methodology (equal), software (equal), validation (equal), visualization (equal), writing – original draft (equal), writing – review and editing (equal). **Coşkun Tez:** conceptualization (equal), data curation (equal), formal analysis (equal), investigation (equal), methodology (equal), supervision (equal), validation (equal), visualization (equal), writing – original draft (equal), writing – review and editing (equal). **Osman İbiş:** conceptualization (equal), data curation (equal), formal analysis (equal), funding acquisition (equal), investigation (equal), methodology (equal), project administration (equal), resources (equal), software (equal), supervision (equal), validation (equal), visualization (equal), writing – original draft (equal), writing – review and editing (equal).

## Funding

This work was supported by the Research Fund of Erciyes University (Project No.: FBAÜ‐2023–12262).

## Ethics Statement

Sampling of *N. turcicus* was authorized by the General Directorate of Nature Conservation and National Parks, Ministry of Agriculture and Forestry of the Republic of Türkiye (Protocol No: E‐21264211‐288.04‐10,822,834), and laboratory procedures were approved by the Local Ethical Committee for Laboratory Animal Experimentation at Erciyes University (Protocol No: 23/061; April 5, 2023). Specimens of *N. nehringi* were collected under permission No. 72784983–488.04‐23,381 issued by the Republic of Türkiye Ministry of Forestry and Water Affairs, Directorate of Nature Conservation and National Parks, in accordance with decision No. 2015/129, and experimental procedures were approved by the Kafkas University Local Ethics Committee for Animal Experiments (No: 2015/129).

## Conflicts of Interest

The authors declare no conflicts of interest.

## Supporting information


**Figure S1:** Predicted secondary‐structure models of mitochondrial tRNAs in *N. nehringi* , showing canonical cloverleaf conformations and putative structural deviations.

**Figure S2:** Predicted secondary‐structure models of mitochondrial *tRNA*s in *N. turcicus*, showing canonical cloverleaf conformations and putative structural deviations.

**Table S1:** Mitochondrial sequences used for analyses in this study.

## Data Availability

The Turkish *N. turcicus* and *N. nehringi* mitogenome sequencing data are available at NCBI‐ GenBank: PX912127, PX912126.
