# Supplementary figures and images for: Mitogenome Characterization and Phylogenetic Insights Into Blind Mole Rats, Nannospalax nehringi, and N. turcicus, From Türkiye
Source: Ecol Evol. 2026 Jul 10;16(7):e73989. doi: 10.1002/ece3.73989 (PMC13354850; doi:10.1002/ece3.73989)

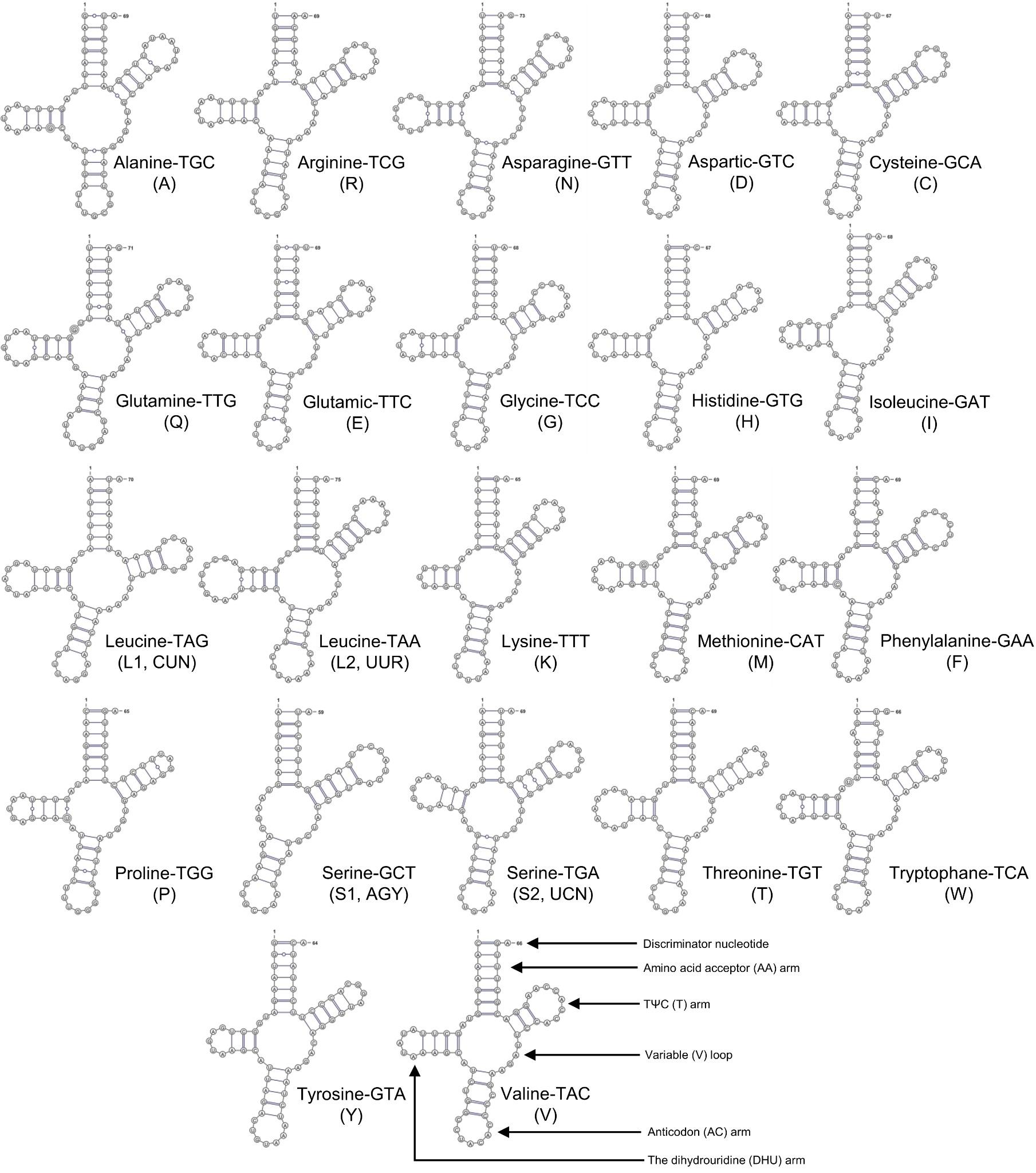

Supplement: Supplementary file 1 — Figure S1: Predicted secondary‐structure models of mitochondrial tRNAs in N. nehringi , showing canonical cloverleaf conformations and putative structural deviations. [file ECE3-16-e73989-s003.jpg]

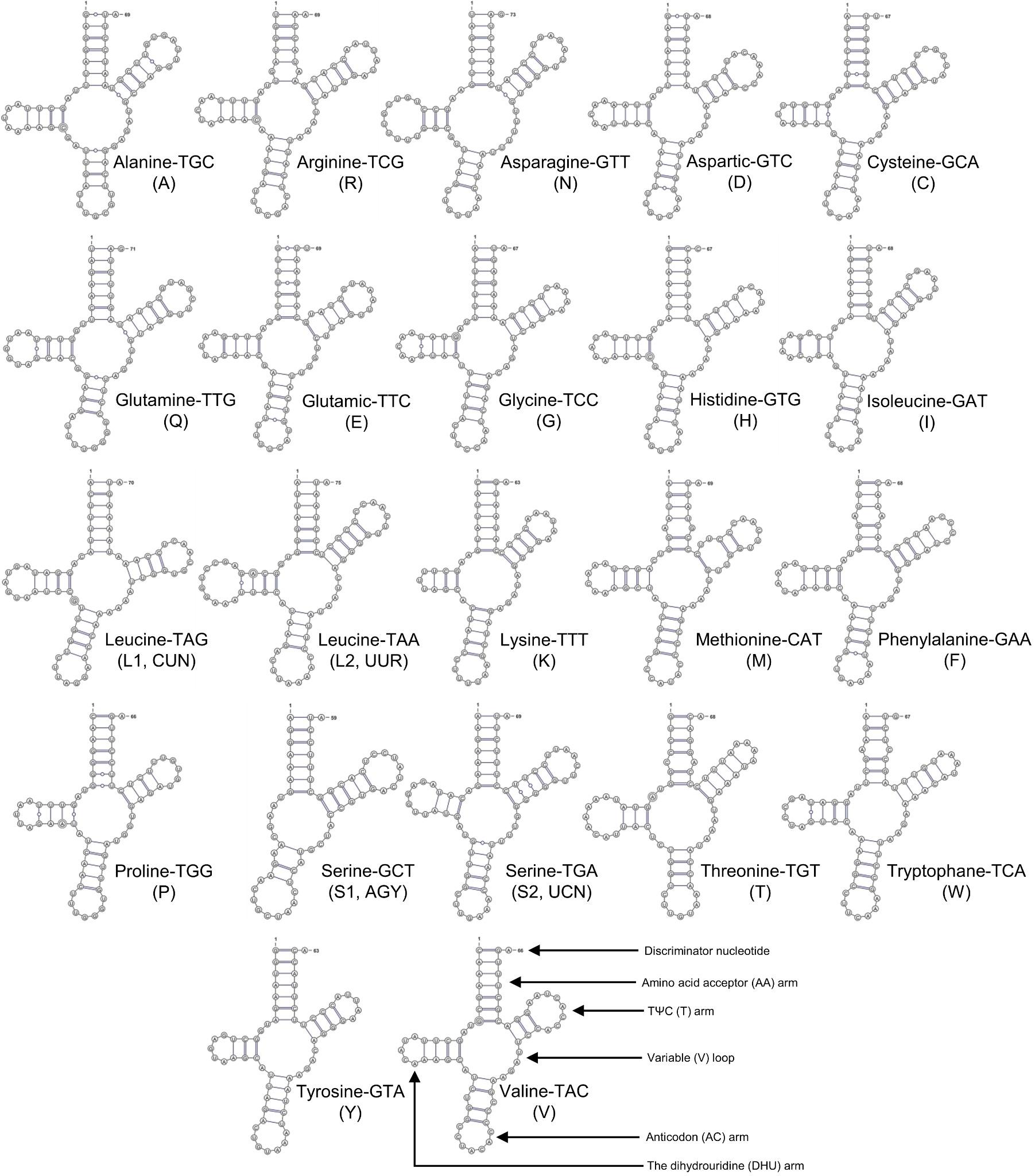

Supplement: Supplementary file 2 — Figure S2: Predicted secondary‐structure models of mitochondrial tRNAs in N. turcicus, showing canonical cloverleaf conformations and putative structural deviations. [file ECE3-16-e73989-s001.jpg]
