# Supplementary material for: Mitogenome Characterization and Phylogenetic Insights Into Blind Mole Rats, Nannospalax nehringi, and N. turcicus, From Türkiye
Source: Ecol Evol. 2026 Jul 10;16(7):e73989. doi: 10.1002/ece3.73989 (PMC13354850; doi:10.1002/ece3.73989)
Supplement: Supplementary file 3 — Table S1: Mitochondrial sequences used for analyses in this study. [file ECE3-16-e73989-s002.docx]

**Table S1.** Mitochondrial DNA sequences used for analyses in this study (N/A: not applicable).

| **NCBI Accession** | **Length of sequence** | **Taxon** | **Subfamily** | **Location** | **Reference** |
| --- | --- | --- | --- | --- | --- |
| ***Complete mitogenome*** |  | | | | |
| JN571130 | 16408 bp | *Nannospalax galili* | Spalacinae | Israel | Hadid et al., 2012 |
| NC_020754 / JN571133 | 16409 bp | *Nannospalax galili* | Spalacinae | Israel | Hadid et al., 2012. |
| JN571129 | 16408 bp | *Nannospalax galili* | Spalacinae | Israel | Hadid et al., 2012 |
| JN571132 | 16408 bp | *Nannospalax galili* | Spalacinae | Israel | Hadid et al., 2012 |
| JN571131 | 16408 bp | *Nannospalax galili* | Spalacinae | Israel | Hadid et al., 2012 |
| NC_020757 / JN571138 | 16402 bp | *Nannospalax golani* | Spalacinae | Israel | Hadid et al., 2012 |
| JN571134 | 16403 bp | *Nannospalax golani* | Spalacinae | Israel | Hadid et al., 2012 |
| NC_020756 / JN571137 | 16408 bp | *Spalax carmeli / Nannospalax carmeli* | Spalacinae | Israel | Hadid et al., 2012 |
| JN571136 | 16409 bp | *Nannospalax judaei* | Spalacinae | Israel | Hadid et al., 2012 |
| NC_020755 / JN571135 | 16406 bp | *Nannospalax judaei* | Spalacinae | Israel | Hadid et al., 2012 |
| NC_005315 / AJ416891 | 16408 bp | *Nannospalax ehrenbergi* | Spalacinae | N/A | Reyes et al., 2004 |
| NC_026915 / KP724691 | 16436 bp | *Myospalax aspalax* | Myospalacinae | N/A | Yuan et al., 2016 |
| NC_026034 / JX014234 | 16360 bp | *Myospalax psilurus* | Myospalacinae | N/A | Li et al., 2016a |
| NC_045904 / MH884481 | 16369 bp | *Eospalax fontanierii* | Myospalacinae | N/A | Cai et al., 2019 |
| NC_047427 / MH933740 | 16355 bp | *Eospalax rufescens* | Myospalacinae | China | Song et al., 2020. |
| NC_048987 / MH891800 | 16350 bp | *Eospalax smithii* | Myospalacinae | China | Cai et al., 2020 |
| NC_018535 / JN544420 | 16377 bp | *Eospalax rothschildi* | Myospalacinae | N/A | Direct Submission |
| NC_021129 / KC514112 | 16354 bp | *Eospalax cansus* | Myospalacinae | China | Su et al., 2013 |
| NC_018098 / JN540033 | 16351 bp | *Eospalax baileyi* | Myospalacinae | China | Liu et al., 2011 |
| NC_058275 / MW751806 | 16646 bp | *Tachyoryctes macrocephalus* | Rhizomyinae | Ethiopia | Reuber et al., 2021 |
| NC_026124 / KM434232 | 16564 bp | *Rhizomys sinensis* | Rhizomyinae | China | Xu et al., 2016 |
| NC_039104 / MF405074 | 16672 bp | *Rhizomys sumatrensis* | Rhizomyinae | China | Xu et al., 2018 |
| NC_021478 / KC789518 | 16575 bp | *Rhizomys pruinosus* | Rhizomyinae | China | Zhao et al., 2014 |
| MG193909 | 16579 bp | *Rhizomys pruinosus* | Rhizomyinae | China | Zhao et al., 2014 |
| NC_023263 / KF425526 | 16351 bp | *Meriones unguiculatus* | Gerbillinae | China | Li et al., 2016b |
| ***Mitochondrial Cyt b*** |  | | | | |
| JX451854 | 1139 bp | *Nannospalax nehringi* | Spalacinae | Türkiye | Krystufek et al., 2012 |
| MH300078- UNVERIFIED | 1140 bp | *Nannospalax leucodon* | Spalacinae | Türkiye | Matur et. al., 2019 |
| MH300079 | 1140 bp | *Nannospalax leucodon* | Spalacinae | Türkiye | Matur et. al., 2019 |
| OR751035 | 1140 bp | *Nannospalax turcicus* | Spalacinae | Türkiye | Németh et al., 2024 |
| OR751035 | 1140 bp | *Nannospalax turcicus* | Spalacinae | Türkiye | Németh et al., 2024 |

**References**

Cai, Z., Zhang, Y., Gao, H., Song, P., Zhang, J., Zhang, T., 2019. The complete mitochondria lgenome of Chinese zokor (*Eospalax fontanierii*). Mitochondrial DNA Part B 4, 153–154.

Cai, Z., Zhang, J., Qiao, P., Qing, W., Zhang, T., 2020. Next generation sequencing yields the complete mitogenome of Smith’s zokor (*Eospalax smithii*). Mitochondrial DNA B Resour 5, 2109–2110. https://doi.org/10.1080/23802359.2020.1765211

Hadid Y, Németh A, Snir S, Pavlíček T, Csorba G, Kázmér M, Major A, Mezhzherin S, Rusin M, Coşkun Y, Nevo E. Is evolution of blind mole rats determined by climate oscillations? PLoS One. 2012;7(1):e30043. doi: 10.1371/journal.pone.0030043.

Krystufek,B., Ivanitskaya,E., Arslan,A., Arslan,E. and Buzan,E.V. 2012. Evolutionary history of mole rats (genus Nannospalax) inferred from mitochondrial cytochrome b sequence. Biol. J. Linn. Soc. Lond. 105 (2), 446-455

Li, Y., Lu, J., & Wang, Z. 2016a. Complete mitochondrial genome of Manchurian Zokor (*Myospalax psilurus*). Mitochondrial DNA Part A, 27(2), 1461–1462. https://doi.org/10.3109/19401736.2014.953093

Li, C.L., Du, X.Y., Gao, J., Wang, C., Guo, H.G., Dai, F.W., Sa, X.Y., An, W., Chen, Z.W., 2016b. Phylogenetic analysis of the Mongolian gerbil (*Meriones unguiculatus*) from China based on mitochondrial genome. Genet Mol Res 15. https://doi.org/10.4238/gmr.15037703

Liu, Z., Li, Y., Shi, F., Lu, J., Li, M., Wang, Z., 2011. Mitochondrial genome of Plateau zokor *Myospalax baileyi*. Mitochondrial DNA 22, 174–175. https://doi.org/10.3109/19401736.2011.636438

Matur, F., Yanchukov, A., Colak, F., Sözen, M., 2019. Two major clades of blind mole rats (*Nannospalax* sp.) revealed by mtDNA and microsatellite genotyping in Western and Central Turkey. Mammalian Biology 94, 38–47.

Németh A, Mizsei E, Laczkó L, Czabán D, Hegyeli Z, Lengyel S, Csorba G, Sramkó G. Evolutionary history and systematics of European blind mole rats (Rodentia: Spalacidae: Nannospalax): Multilocus phylogeny and species delimitation in a puzzling group. Mol Phylogenet Evol. 2024 Jan;190:107958. doi: 10.1016/j.ympev.2023.107958.

Reuber, V.M., Rey-Iglesia, A., Westbury, M.V., Cabrera, A.A., Farwig, N., Skovrind, M., Šumbera, R., Wube, T., Opgenoorth, L., Schabo, D.G., Lorenzen, E.D., 2021. Complete mitochondrial genome of the giant root-rat (*Tachyoryctes macrocephalus*). Mitochondrial DNA B Resour 6, 2191–2193. https://doi.org/10.1080/23802359.2021.1944388

Reyes, A., Gissi, C., Catzeflis, F., Nevo, E., Pesole, G., Saccone, C., 2004. Congruent mammalian trees from mitochondrial and nuclear genes using Bayesian methods. Mol Biol Evol 21, 397–403. https://doi.org/10.1093/molbev/msh033

Song P, Gao H, Jiang F, Zhang T, Cai Z. Characteristics of the mitochondrial genome of Qinling zokor (*Eospalax rufescens*). Mitochondrial DNA B Resour. 2020 May 27;5(3):2161-2162. doi: 10.1080/23802359.2020.1768919

Su, J., Wang, J., Hua, L., Gleeson, D., Ji, W., 2013. Complete mitochondrial genome of the Gansu zokor, *Eospalax cansus* (Rodentia, Spalacidae). Mitochondrial DNA 24, 651–653. https://doi.org/10.3109/19401736.2013.772166

Xu, Y., Liu, X., Tu, F., 2016. Complete mitochondrial genome of Chinese bamboo rat, *Rhizomys sinensis* and species divergence comparison. Mitochondrial DNA A DNA Mapp Seq Anal 27, 1773–1774. https://doi.org/10.3109/19401736.2014.963806

Xu, Y., Wen, B., Zhu, K., Du, Z., Bai, X., 2018. The complete mitochondrial genome of *Rhizomys sumatrensis* (Rodentia: Spalacidae) and its phylogenetic implications. Conservation Genet Resour 10, 685–688. https://doi.org/10.1007/s12686-017-0901-3

Yuan, S., Lu, Z., Wu, X., Fu, H., Bao, D., Malqin, H., Yang, S., 2016. Complete mitochondrial genome of *Myospalax aspalax*  (Rodentia, Spalacidae). Mitochondrial DNA Part A 27, 4250–4251.

Zhao, F., Zhang, T., Su, J., Nevo, E., Lin, G., 2014. Mitochondrial genome of bamboo rat *Rhizomys pruinosu*s. Mitochondrial DNA 25, 381–382. https://doi.org/10.3109/19401736.2013.809434
